# Lutetium-177 Prostate-Specific Membrane Antigen-617 Treatment in Metastatic Castration-Resistant Prostate Adenocarcinoma: Results of Single-Center Experience

**DOI:** 10.5152/eurasianjmed.2023.0055

**Published:** 2023-06-01

**Authors:** Adem Maman

**Affiliations:** 1 Department of Nuclear Medicine, Atatürk University, Faculty of Medicine, Erzurum, Turkey

**Keywords:** Prostate cancer, 177Lu-PSMA-617, 68Ga-PSMA-11 PET/CT, PSA, pain score, SUVmax

## Abstract

**Objective:**

Lutetium-177 prostate-specific membrane antigen-617 is a novel alternative therapeutic option in metastatic castration-resistant prostate cancer, especially useful for patients who do not respond to standard therapy methods. The aim of this study was to define the efficacy and safety profile of lutetium-177 prostate-specific membrane antigen-617 treatment in a group of patients with metastatic castration-resistant prostate cancer.

**Materials and Methods:**

Study group included 34 men with metastatic castration-resistant prostate cancer (median, 69.6 ± 7.7 years) who were treated with lutetium-177 prostate-specific membrane antigen-617 therapy (22/34; 4 courses, 12/34; 2 courses). Patients were evaluated by physical examination, Eastern cooperative oncology group performance status, gallium-68 prostate-specific membrane antigen positron emission tomography/computed tomography, brief pain inventory-short form questionnaire, biochemical tests, and complete blood counts. Treatment response and adverse effects were examined by brief pain inventory scores, SUV_max_ values, biochemical tests, and complete blood counts. Independent variables were analyzed statistically (significance; *P* < .05).

**Results:**

The Eastern cooperative oncology group performance was grade 0 in 5/34 (14.7%), grade 1 in 25/34 (73.5%), and grade 2 in 4/34 (11.8%) patients. Distribution of patient numbers according to brief pain inventory scores (score: <1, scores: 1-4, and scores: 5-10) was 2, 10 and 22 at the beginning, 6, 16 and 12 after the second course, and 10, 10 and 2 after the fourth course of treatment, respectively. Serum prostate-specific antigen decreased in 15 of 22 patients (68%) (*P* < .05). Before and after the treatment, we found a substantial decrease in SUV_max_ values (22.3 vs. 11.8, *P* < .001) and brief pain inventory scores (score ≥ 5; 22/34 pts vs. 0/22 pts). The counts of white blood cells (*P *< .05), hemoglobin (*P *< .05), and thrombocytes (*P* = .001) were all significantly lower at the conclusion of the therapy. The most important adverse events were severe leukopenia (1/34 pts; 2.29 × 10^3^/µL) and thrombocytopenia (3/34 pts; 32 000, 36 000, 32 000 × 10^6^/L).

**Conclusion:**

We found that lutetium-177 prostate-specific membrane antigen-617 therapy is a promising treatment method for metastatic castration-resistant prostate cancer patients who are unresponsive to conventional therapy, according to our biochemical, positron emission tomography/computed tomography, and pain score outcomes.

**Main Points**

- Lutetium-177 prostate-specific membrane antigen-617 (^177^Lu-PSMA-617) therapy is an important treatment option for metastatic castration-resistant prostate cancer (mCRPC) patients who do not respond to conventional treatment protocols.
- ^177^Lu-PSMA-617 treatment significantly reduces the pain that negatively affects the quality of life in patients with metastatic prostate cancer.
- ^177^Lu-PSMA-617 treatment appears to be safe in patients with mCRPC with a low side effect profile.

## Introduction

Prostate adenocarcinoma is the second commonest cancer worldwide and one of the leading causes of cancer death. It still has significant morbidity and mortality despite diagnostic and therapeutic advances.^1,2^ Androgen deprivation therapy (ADT) is the gold standard method for patients with prostate cancer. In spite of the high initial response rates, cancer treatment with ADT is of limited duration; many men eventually develop progressive disease, so-called metastatic castration-resistant prostate cancer (mCRPC) following ADT.^3^ Since the approval of docetaxel as the first-line chemotherapy in 2004, several new life-prolonging systemic therapies such as abiraterone, enzalutamide, cabazitaxel, and ^223r^adium have become available for mCRPC patients. Despite these treatments, many patients have progressed to advanced cancer stages despite new treatment modalities. Today, effective therapeutic alternatives are needed to control disease-related symptoms and to improve quality of life.

Lutetium-177 prostate-specific membrane antigen (^177^Lu-PSMA-617) has become a potent treatment agent thanks to the increased expression of PSMA in most men with mCRPC.^4,5^ It has been reported that ^177^Lu-PSMA-617 treatment is valuable in providing biochemical and symptomatic pain control and improving quality of life in mCRPC patients.^5^

Prostate-specific membrane antigen, also called folate hydrolase I or glutamate carboxypeptidase II, is expressed at high levels in prostatic adenocarcinoma cells. It has been reported that there is a significant increase in PSMA levels of patients who have either high-grade or castration-resistant cancers. Prostate-specific membrane antigen represents an excellent biomarker for both imaging and treatment of prostate cancer and so this topic has become the focus of extensive research. Some tissues have varying degrees of PSMA expression, including prostate epithelium, small intestine, renal tubules, and salivary glands.^6^

Prostate-specific membrane antigen is a type II transmembrane protein with 2 monomers and corresponding intracellular transmembrane and extracellular domains that are enzymatically active proteins in homodimeric form.^7^ In ligand binding, PSMA undergoes clathrin-mediated endocytosis.^8^ Identification of the substrate and binding site has spurred the development of urea-based high-affinity PSMA inhibitors with favorable biodistribution and high tumor-to-background uptake rates.^6^ Lutetium-177 prostate-specific membrane antigen-617 synthesis was originally developed by the German Cancer Research Center (DKFZ, DeutschesKrebsforschungszentrum) in collaboration with University Hospital Heidelberg. It is a small molecule inhibitor that binds to PSMA with high affinity. The short-range 1 mm path length of the beta particle emitted by ^177^Lu ensures effective delivery of radiation to tumoral tissue while minimizing damage to surrounding normal tissues.^9^

The aim of this retrospective study was to report our results and confirm the efficacy and side effect profile of ^177^Lu-PSMA-617 treatment in mCRPC patients.

## Materials and Methods

### Study Group

A total of 42 consecutive patients, between February 2017 and November 2021, were referred to the Department of Nuclear Medicine of Atatürk University Medical Faculty Hospital. All patients were discussed by an interdisciplinary tumor board for ^177^Lu-PSMA-617 therapy recommendation due to mCRPC. The study protocol conforms to the Declaration of Helsinki and was approved by the local ethics committee (decision date: November 25, 2021; decision number: 08-26). Since this study was a retrospective study, informed consent form could not be obtained from the patients. After evaluation according to exclusion criteria, the final study group consisted of 34 mCRPC patients (age range; 69.6 ± 7.7 years) who underwent ^177^Lu-PSMA-617 therapy.

### Pre-Evaluation and Exclusion Criteria of Patients

At the time of hospitalization, all patients with mCRPC who were candidates for ^177^Lu-PSMA-617 therapy were subjected to a physical examination by an experienced medical doctor, and necessary tests were performed for preliminary evaluation. Patients were graded by the Eastern cooperative oncology group (ECOG) performance status and Brief Pain Inventory (BPI) score according to previously published criteria.^10^ Routinely measured laboratory parameters in each patient included complete blood counts and biochemistry tests [liver function enzymes, serum creatinine, and prostate-specific antigen (PSA) levels]. Each patient underwent a gallium-68 prostate-specific membrane antigen positron emission tomography/computed tomography (^68^Ga-PSMA-11 PET/CT) prior to treatment to demonstrate the presence of PSMA over-expression in their lesions. 

We used some exclusion criteria and 8 patients were excluded from this study. The criteria for blood picture are as follows: liver enzymes more than 5 times the upper limit, total white blood cell (WBC) count less than 3 × 10^9^/L, platelet count less than 75×10^9^/L, and hemoglobin less than 8 g/dL. We excluded 4 patients (n = 4) due to low platelet counts. The other 4 excluded patients (n = 4) had metastases on CT but no PSMA expression on ^68^Ga-PSMA-11 PET/CT.

### Lutetium-177 prostate-specific membrane antigen-617 therapy

We followed a previously published ^177^Lu-PSMA-617 treatment protocol for all patients.^6^ We applied 4 courses of treatment to 22 of 34 (64.7%) patients. We had to apply 2 courses of treatment in 12 of 34 patients due to the following reasons; six patients refused to continue the next courses of the treatmente clinicians decided to terminate the treatment in four patients, two patients died due to another concomitant disease before the third course of the treatment. The interval between each course of treatment was 6-8 weeks. During each administration, patients received an infusion of 1 L of normal saline at 300 mL/h, 30 min before ^177^Lu-PSMA-617 administration with an average dose of 7315 ± 573 MBq. We did not use a special protection method for the salivary glands.

## Evaluation of Treatment Response

A rate of change in BPI score, serum PSA, and lesion SUV_max_ values obtained before and after administration of ^177^Lu-PSMA-617 was examined to evaluate the treatment response. Follow-up BPI score assessments were repeated 2 times; 45 days after the fourth course of the treatment in 34 patients and 1 month after the fourth course of the treatment in 22 patients. Serum PSA measurements and ^68^Ga-PSMA-11 PET/CT were routinely performed in each patient 1 month after the last course of ^177^Lu-PSMA-617 administration. 

### Evaluation of Side Effects

All patients were evaluated in order to define treatment-related side effects. They were questioned for the presence of newly developed symptoms after each course administration. They were examined by complete blood counts and biochemistry tests 1 month after the last course of treatment and test results were analyzed for change before and after ^177^Lu-PSMA-617 therapy. Toxicity-attributed side effects and hematologic changes were documented according to version 4.0 of the Common Toxicity Criteria for Adverse Events.

### Statistical Analysis

Statistical analyses were performed using the International Business Machines’ Statistical Package for the Social Sciences Statistics for Windows, Version 22.0 (IBM Corp., Armonk, NY, USA). The variables were investigated using visual (histograms, probability plots) and analytic methods (Kolmogorov–Smirnov/Shapiro–Wilks test) to determine whether or not they are normally distributed. Data with normal distribution are given as mean ± standard deviation (SD), and the data whose distribution was not normal are given as median (interquartile range). After checking the normality distribution of scale variables, independent samples were compared with appropriate significance tests (e.g., the Mann–Whitney *U* test, Kruskal–Wallis H test). The results with *P* < .05 were considered statistically significant.

## Results

The characteristics of the patients included in the study are given in Table 1. The mean age of 34 patients was 69.6 ± 7.7 years. Eastern cooperative oncology group performance status of the patients was grade 0 in 5/34 patients (14.7%), grade 1 in 25/34 patients (73.5%), and grade 2 in 4/34 patients (11.8%). All patients had bone metastases, whereas 14/34 patients (41.2%) had lymph node metastases. In addition to ^177^Lu-PSMA-617 administration, 10/34 patients (29.4%) received a standard chemotherapy regimen and 12/34 patients (35.3%) received a standard chemotherapy regimen + second-generation hormone therapy. Around 12 of 34 patients (35.3%) did not receive standard chemotherapy or second-generation hormone therapy prior to ^177^Lu-PSMA-617 treatment.

The BPI score values of 34 mCRPC patients before and after treatment are given in Table 2.

The distribution of patient numbers according to BPI scores (score:<1, score:1-4 and score:5-10) were 2, 10 and 22 at the beginning, 6, 16 and 12 after the second course, and 10, 10 and 2 after fourth course of treatment, respectively. No acute event development was observed during the treatment applications. Following 177Lu-PSMA-617 administration, patients' bone pain and quality of life improved progressively. Initially, 32 of 34 patients (94.1%) had pain complaints. After the second course of treatments, the number of patients with pain decreased from 32 to 25 (82.3%). While the number of patients who experienced moderate/severe pain at the beginning was 22, 10 patients had moderate/severe pain after 2 courses of 177Lu-PSMA-617 treatment (decrease from 64.7% to 35.3%). In addition to these, it is reported that 22/34 patients who completed 4 courses of treatment. Among these, 10/22 patient (45.5%) no longer complained of pain. Only 2/22 patient (9%) had moderate pain while 10/22 patients (45.5%) had mild pain.

**Figure 1.**
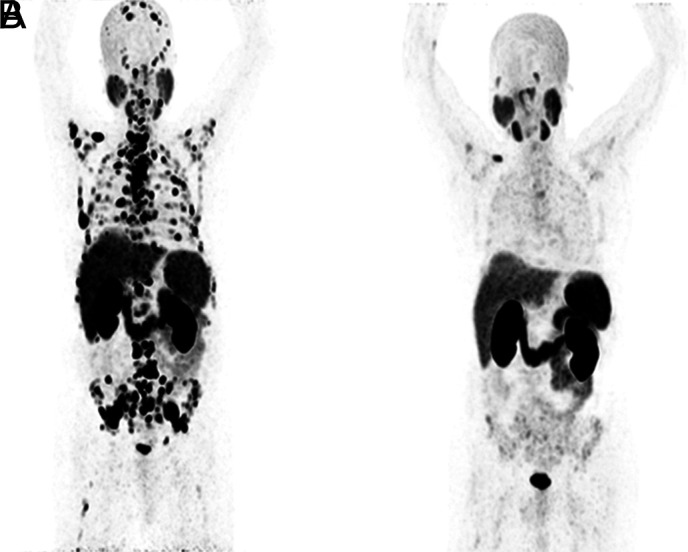
Baseline (A) and follow-up (B) after 4 cycles ^68^Ga-PSMA-11 PET/CT of a patient with mCRPC, who was treated with 7315 ± 573 MBq ^177^Lu-PSMA-617. Prostate-specific antigen (PSA) response was as follows: 107.8 ng/mL (baseline) and 2.92 ng/mL (after 4 cycles). ^68^Ga-PSMA-11 PET/CT, gallium-68 prostate-specific membrane antigen positron emission tomography/computed tomography;^ 177^Lu-PSMA-617, lutetium-177 prostate-specific membrane antigen-617; mCRPC, metastatic castration-resistant prostate cancer.

Table 3 summarizes the results of biochemical markers, complete blood counts, and ^68^Ga-PSMA-11 PET/CT-derived semiquantitative SUV_max_ values of the patients and the comparisons of pre- and post-treatment values to assess treatment response and side effects. When we compared pre- and post-treatment PSA levels of the patients, we found a statistically significant difference between these 2 data sets (*P* < .05, 115 vs. 24 ng/mL, Figure 1). After the fourth course of ^177^Lu-PSMA-617 therapy, a PSA decline was detected in 15 of 22 patients (68.1%). Thirteen of these 22 patients (59%) had a decrease of more than 50%, and there was more than 80% reduction in 9 of them (40.9%). In agreement with the decreasing PSA values, we found a statistically significant difference between pre- and post-treatment bone SUV_max_ values (*P* < .001) and a distinct decrease in median SUV_max_ values (22.2 vs. 11.8). Gallium-68 prostate-specific membrane antigen positron emission tomography/computed tomography images of a patient before and after 4 sessions of ^177^Lu-PSMA-617 treatment are given in Figure 1.

**Figure 2.**
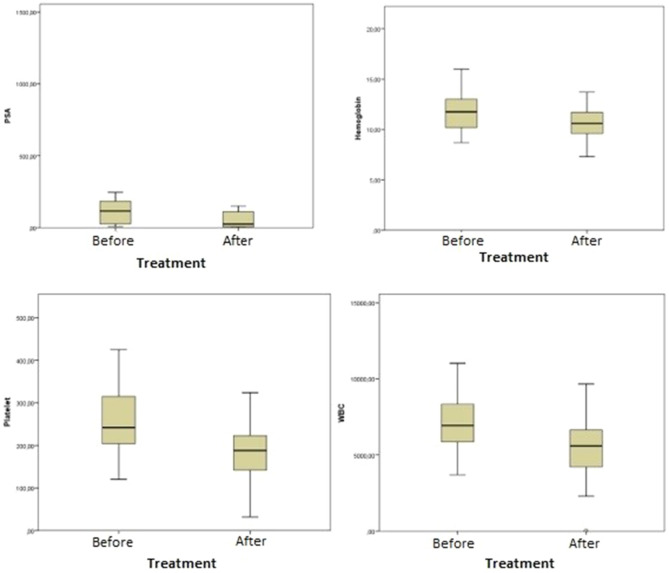
Box plots of prostate-specific antigen (top left), platelet (bottom left), hemoglobin (top right), and white blood cells (bottom right) of metastatic castration-resistant prostate cancer patients before and after lutetium-177 prostate-specific membrane antigen therapy-617 (^177^Lu-PSMA-617).

After ^177^Lu-PSMA-617 treatment, no significant change was detected in serum creatinine and calcium levels of the patients (*P* > .05). A statistically significant decrease was found in the WBC, hemoglobin, and platelet counts of the patients after ^177^Lu-PSMA-617 treatment (*P* values; <.05, <.05, =.001, respectively) (Table 3 and Figure 2). In addition, severe leukopenia (2.29 10^3^/µL) was observed in 1 patient and severe thrombocytopenia (32 000, 36 000, 32 000 10^6^/L) developed in 3 patients. 

## Discussion

Prostate cancer is one of the most common types of human urogenital system malignancies. It still has serious morbidity and mortality despite the use of new treatment protocols and advanced diagnostic imaging methods. Androgen deprivation therapy is positioned as the first-line application in the treatment algorithm of prostate cancer. A combination of chemotherapy, radiotherapy, and a second-generation anti-androgen drug is often preferred in patients with advanced prostate cancer. In recent years, ^177^Lu-PSMA-617 has been used more frequently as an alternative or complementary treatment option in advanced disease, especially for mCRPC patients.^1,2^

Prostate-specific antigen is a useful biomarker approved by US Food and Drug Administration for diagnosing and monitoring prostate cancer. It is especially useful in the follow-up of patients with advanced disease and it correlates well with their clinical status. According to the results of a pooled meta-analysis studied on 10 different studies which are investigating the efficacy of ^177^Lu-PSMA-617 therapy, there was a decrease in PSA level in 165 of 238 patients (69.3%).^11^ This meta-analysis also confirmed our results (68%). Extreme low and high efficacy values were also observed. However, some studies have reported lower and higher rates. In the study by Ahmadzadehfar et al.^12^ a decrease in PSA level after ^177^Lu-PSMA-617 therapy was found to be 79.1%, while Kratochwil^9^ found it to be 72%. Rahbar et al^13^, on the other hand, measured the decrease in PSA as 59.7%.

According to meta-analysis by Emmett et al.^14^ hematological side effects are common and significant, especially for bone metastasis. In their analysis, hemoglobin levels ranged from 10% to 32%, platelet counts ranged from 0% to 25%, and WBC counts ranged from 3% to 15%. Our study is compatible with the meta-analysis of Emmett in terms of hematological side effects. In our study, hemoglobin level, platelet, and WBC count decreased by 8.94%, 29%, and 18%, respectively. This side effect is mostly observed either in grade 1 or grade 2. However, severe side effects could be reported in patients who take chemotherapy before the ^177^Lu PSMA. 

The significant decrease in PSA value, which is accepted as the most important biomarker of prostate cancer, can predict that patients respond positively to ^177^Lu-PSMA-617 treatment. In our study, ^177^Lu-PSMA-617 treatment was administered to patients who progressed despite chemotherapy and second-generation anti-androgen therapy and to patients who were unsuitable or did not accept this treatment. In our series, when we evaluate PSA and bone SUV_max_ values of patients, a significant decrease was found in the PSA levels (*P* < .05), and a significant decrease was observed in the SUV_max_ values of the patients (*P* < .001) according to the ^68^Ga-PSMA-11 PET/CT scores obtained before and after the treatment. This significant decrease in SUV_max_ values is another good indicator of response to the treatment. When these 2 parameters were integrated, it was seen that the patients have a positive response to this alternative treatment. According to our survey on pain scores, it was reported that patients felt severe bone pain before and after the therapy. A remarkable decrease was observed in many patients even after the administration of the first dose. Significant pain reduction was observed in 27 (79.41%) of 34 patients; although there was no decrease in PSA level in 3 patients, a decline in pain score was also observed. 

In all patients, WBC, calcium, hemoglobin, platelet, and creatinine levels were followed; even if some of those values decreased during therapy, the values were within normal limits. It was reported that only 1 patient developed severe leukopenia and 3 patients developed severe thrombocytopenia. Taking into account all mentioned findings and the severity of present side effects, we emphasized that the ^177^Lu-PSMA-617 treatment does not have a serious side effect profile, as well as it has promising results and remarkable improvements in the patient's quality of life. 

This study has some limitations as it was carried out with a limited number of patients in a small group. For more precise results, the patient group should be enlarged. Another limitation of the study is that we could not complete the standard 4 courses treatment regimen in all patients. This expectation is difficult to meet due to patient compliance, other treatment options, co-morbidities, and social reasons. Nevertheless, we think that our findings still remain reliable since most of the patients in the study group (22/34; 64.7%) met this requirement.

In conclusion, ^177^Lu-PSMA-617 therapy is an important treatment option for mCRPC patients who do not respond to conventional treatment protocols. It has a low side effect profile and can improve patients’ quality of life thanks to its therapeutic effect on metastatic lesions.

## Figures and Tables

**Table 1.** Demographical and Clinical Features Before ^177^Lu PSMA of 34 Patients with mCRPC Treatment

Parameters	Values
**Number of patients (n)**	34
**Age (mean ± SD)**	69.6 ± 7.7
**ECOG Index (n/%)**	
Grade 0	5/14.7
Grade 1	25/73.5
Grade 2	4/11.8
**PSMA-RLT before (n / %)**	
Chemotherapy	10/29.4
Chemo-hormonal therapy	12/35.3
**Metastatic lesion (n / %)**	
Bone	34/100
Lymph node	14/41.2

ECOG Index, Eastern Cooperative Oncology Group Index; PSMA-RLT, prostate-specific membrane antigen directed radioligand therapy.

**Table 2.** Distribution of mCRPC Patients’ BPI Scores at the B eginning (n = 34) and After the Second Course (n = 34) and Fourth Course (n = 22) of 177Lu-PSMA-617 Therapy

	Initial	After the Second Course of Treatment	After the Fourth Course of Treatment
	n (%)	n (%)	n (%)
**No pain **(score: <1)	2 (5.8)	6 (17.6)	10 (45.5)
**Mild pain **(scores: 1-4)	10 (29.5)	16 (47.1)	10 (45.5)
**Moderate to severe pain **(scores: 5-10)	22 (64.7)	12 (35.3)	2 (9)

BPI, brief pain inventory; mCRPC, metastatic castration-resistant prostate cancer.

**Table 3.** Comparison of Pre- and Post-treatment Values for Biochemical Markers, Complete Blood Counts, and PET-Derived Semiquantitative SUV_max_ Index of mCRPC Patients

Parameters	Pre-treatment Value	Post-treatment Value	*P*
Creatinine (mg/dL)	0.85 (0.73-1.1)	0.78 (0.54-1.01)	NS
WBC (10^3^/µL)	7.2 ± 2.2	5.9 ± 2.1	<.05
Hemoglobin (g/dL)	11.8 ± 1.9	10.8 ± 1.9	<.05
Platelet (10^3^/mm^3^)	248 ± 75	176 ± 79	.001
Calcium (mg/dL)	9.1 (8.7-9.6)	8.9 (8.4-9.3)	NS
Bone SUV_max_	22.3 (13.7-35.6)	11.8 (5.8-14.5)	<.001
PSA (ng/mL)	115 (24-188)	24 (4.5-115)	<.05

mCRPC, metastatic castration-resistant prostate cancer; NS, not significant (*P* > .05); PSA, prostate-specific antigen; WBC, white blood cells.
